# The Impact of Weight Gain During HIV Treatment on Risk of Pre-diabetes, Diabetes Mellitus, Cardiovascular Disease, and Mortality

**DOI:** 10.3389/fendo.2018.00705

**Published:** 2018-11-27

**Authors:** Shejil Kumar, Katherine Samaras

**Affiliations:** ^1^St George Clinical School, University of New South Wales, Sydney, NSW, Australia; ^2^Department of Endocrinology, St Vincent's Hospital, Sydney, NSW, Australia; ^3^Diabetes and Metabolism Program, Garvan Institute of Medical Research, Sydney, NSW, Australia; ^4^St Vincent's Clinical School, University of New South Wales, Sydney, NSW, Australia

**Keywords:** HIV, cART, weight gain, obesity, diabetes, cardiovascular disease (CVD), mortality

## Abstract

Since the introduction of combined antiretroviral therapy (cART) and more effective treatments for AIDS, there has been a dramatic shift from the weight loss and wasting that characterised HIV/AIDS (and still does in countries where cART is not readily available or is initiated late) to healthy weight, or even overweight and obesity at rates mirroring those seen in the general population. These trends are attributable to several factors, including the “*return to health*” weight gain with reversal of the catabolic effects of HIV-infection following cART-initiation, strategies for earlier cART-initiation in the course of HIV-infection which have prevented many people living with HIV-infection from developing wasting, in addition to exposure to the modern obesogenic environment. Older cART regimens were associated with increased risk of body fat partitioning disorders (lipodystrophy) and cardiometabolic complications including atherothrombotic cardiovascular disease (CVD) and diabetes mellitus. Whilst cART now avoids those medications implicated in causing lipodystrophy, long-term cardiometabolic data on more modern cART regimens are lacking. Longitudinal studies show increased rates of incident CVD and diabetes mellitus with weight gain in treated HIV-infection. Abdominal fat gain, weight gain, and rising body mass index (BMI) in the short-term during HIV treatment was found to increase incident diabetes risk. Rising BMI was associated with increased risk of incident CVD, however the relationship varied depending on pre-cART BMI category. In contrast, a protective association with mortality is evident, predominantly in the underweight and in resource-poor settings, where weight gain reflects access to cART and virological suppression. The question of how to best evaluate, manage (and perhaps constrain) weight gain during HIV treatment is of clinical relevance, especially in the current climate of increasingly widespread cART use, rising overweight, and obesity prevalence and growing metabolic and cardiovascular disease burden in people living with HIV-infection. Large prospective studies to further characterise the relationship between weight gain during HIV treatment and risk of diabetes, CVD and mortality are required.

First recognised as a disease in 1981 (1), HIV-infection and acquired immunodeficiency syndrome (HIV/AIDS) is a global epidemic. Data from The Joint United Nations Programme on HIV/AIDS (UNAIDS) shows that HIV-infection currently affects ≈36.7 million people worldwide and has caused 35 million deaths (2). HIV-infection was initially a rapidly fatal condition due to virus-induced immunosuppression and opportunistic infections. Since the introduction of combined anti-retroviral therapy (cART) in the mid-1990s, the narrative of the natural history of HIV-infection has been re-written: effective virological suppression has dramatically improved prognosis and survival evidenced by declining rates of AIDS-related deaths (2). In nations where cART is readily accessible, HIV-infection is now commonly considered a chronic, treatable illness with life expectancy approaching that of the general population (3, 4).

## Paradigm shifts in cart-initiation in the history of HIV-infection

Recently, global efforts have focussed on increasing access to and early initiation of cART to prevent AIDS-related deaths and HIV transmission (5, 6). Over the last 10 years, the World Health Organisation (WHO) has recommended earlier cART-initiation based on CD4^+^ T-lymphocyte counts falling below progressively higher thresholds: ≤200 cells/μL in 2006 (7), ≤350 cells/μL in 2009 (8), and ≤500 cells/μL in 2013 (9). Global strategies to eradicate HIV transmission led the WHO in 2015 to recommend cART-initiation at HIV diagnosis regardless of CD4^+^ T-lymphocyte counts (5). Further, UNAIDS launched the “90-90-90” targets in 2014 aiming to end the AIDS epidemic by 2030: 90% of people living with HIV-infection knowing their status, 90% receiving sustained cART and 90% of cART-recipients achieving viral suppression by 2020 (6). It is estimated that if these targets are met by 2020, the number of cART-recipients worldwide would increase from 20.9 million (2) to ≈30 million (10, 11).

## The metabolic complications of rising adiposity trends in HIV treatment

The enthusiasm over the improvement in HIV-associated mortality following the introduction of cART has, however, been partly diluted with concerns of cART-associated metabolic complications, including hyperlipidaemia, insulin resistance, and lipodystrophy, which accelerate the onset of type 2 diabetes mellitus (diabetes) and atherothrombotic cardiovascular disease (CVD) (12–14). Lipodystrophy is characterised by peripheral subcutaneous lipoatrophy and central/abdominal lipohypertrophy (12, 15) and has been shown to increase risk of diabetes (13) and myocardial infarction (14) in HIV-treated populations. Whilst earlier cART medications such as nucleoside reverse transcriptase inhibitors (NRTIs) and first-generation protease inhibitors (PIs) were associated with the most clinically evident lipoatrophy and high rates of premature diabetes, longer-term data are lacking on the metabolic consequences of more modern regimens.

## Distinguishing “*return to health*” weight gain from an obesity trajectory

**Figure 1:**
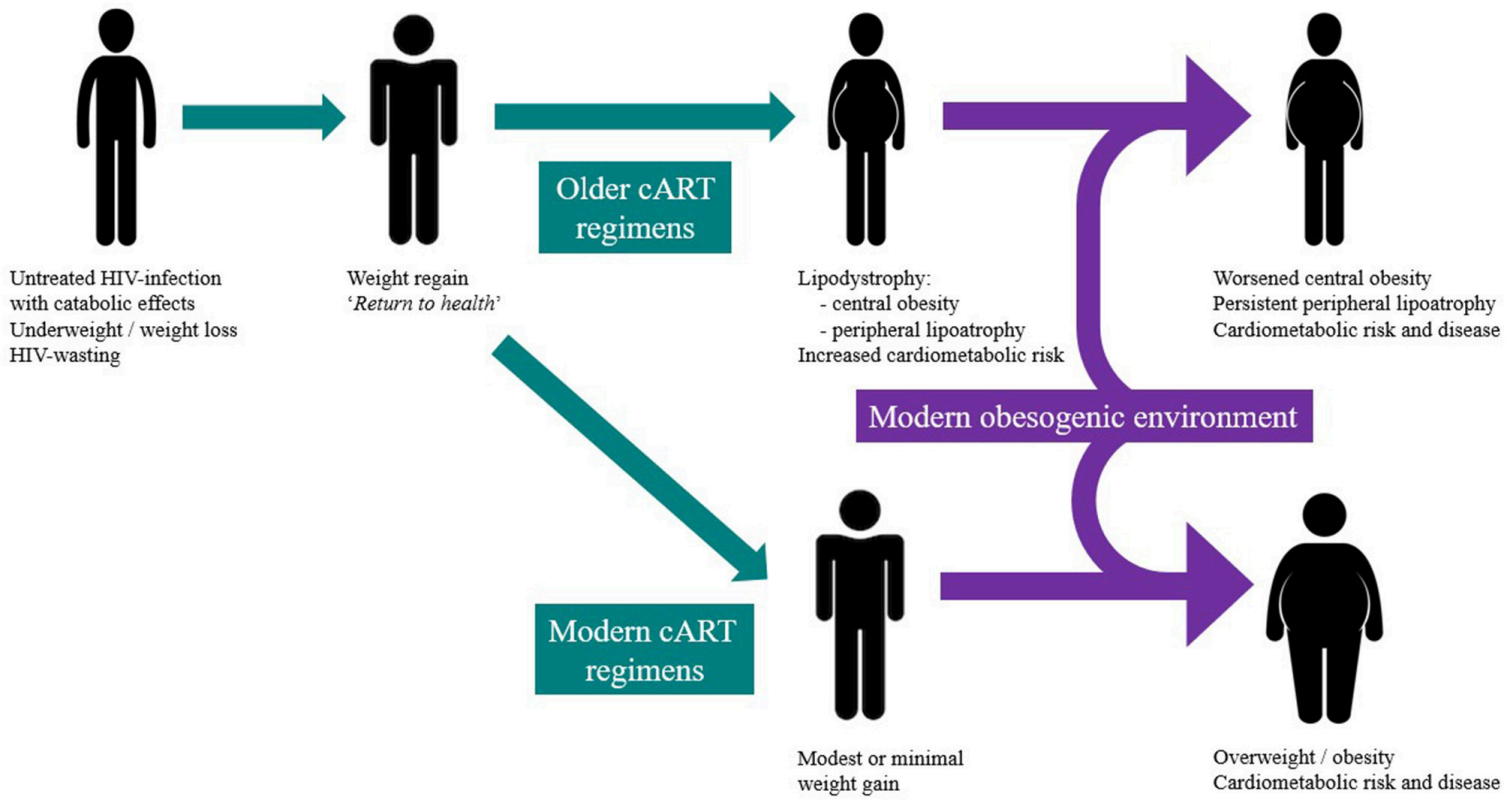
The intersection of the modern obesity epidemic and historical changes in HIV-infection prescription and timing and their contribution to weight gain and redistribution during HIV treatment.

In reviewing the studies that have reported weight gain following cART-initiation, it is important to distinguish weight gain as part of the “*return to health*” phenomenon from clinically undesirable and excessive weight gain or central fat accumulation that places an individual in the overweight and obese category (Figure 1). “*Return to health*” describes the desirable weight gain following resolution of debilitating catabolic infection or illness that restores body fat and protein stores. Earlier cART-initiation has led to fewer people with untreated HIV-infection experiencing the cachectic and wasted state that characterised early clinical experience (16). CART-induced suppression of viral replication and inflammation normalises resting energy expenditure and allows weight regain (16). Therefore, weight gain early after cART-initiation often represents effective viral suppression and CD4^+^ recovery (17) but also restoration of healthy pre-infection weight. However, most studies do not report pre-HIV-infection weight and therefore this important “true” individual baseline measure is lacking. Important data examining body composition changes following recovery from famine or severe catabolic (non-HIV) illness have shown that adipose tissue stores are preferentially restored, as part of the “*return to health*” (18). Robust studies of body composition changes following cART initiation in advanced HIV-infection are lacking, however. One controlled study in the early years of cART use found no changes in body composition between cART recipients over an unspecified time-period, compared to untreated controls with similar CD4 counts and viral load (19). An important distinction of this study was the healthy baseline BMI, indicating the absence of protracted catabolic infection.

Several early small-scale studies have highlighted that after cART-initiation, the degree of weight gain (including measures of abdominal and limb fat mass and lean body mass) is associated with markers of cART efficacy: greater virological suppression, CD4^+^ cell recovery and reduced resting energy expenditure (16, 20–29). More recent and large-scale cART-initiation studies consistently show that the greatest weight gain occurs in those initially underweight with indices of more advanced untreated HIV-infection: the lowest CD4^+^ counts and highest viral load. Pooled data on 760 women and 3,041 men from 3 randomised controlled trials (RCTs) in the United Stated (US) evaluating BMI change in the first 96 weeks after cART-initiation found that initially underweight patients had greatest mean BMI rise (2.53 kg/m^2^), compared to initially normal-weight (1.77 kg/m^2^), overweight (1.17 kg/m^2^) and obese participants (1.16 kg/m^2^) (30). The greatest BMI gain occurred in patients with lower baseline CD4^+^ counts and higher viral load (30), consistent with more advanced infection. An international 48-week study of 246 participants found that the underweight had median 14% increase in BMI (compared to 5% in normal- and overweight participants) and exhibited the greatest decrease in serum CRP levels for each 1.0 kg/m^2^ gain in BMI (31). A prospective 48-week study on 224 participants found that those with virological suppression at 16 weeks post-cART-initiation had greater median weight increase than those who did not (2.1 vs. 0.5 kg) (32). Out of the 143 participants who also had body composition studies, lower baseline CD4 cell counts (< 200 cells/μL) and higher HIV viral load (≥10,000 copies/mL) were associated with modest increases in lean body mass of >1.5 kg and total weight at 16 weeks (32). A recent 96-week study of 269 participants reported mean gains of 4.8 kg in weight, 1.5 kg/m^2^ in BMI and 1.4 kg in lean body mass after cART-initiation (33). Multivariate analyses found that lower baseline CD4^+^ cell counts and higher HIV viral load (again, markers of more advanced infection) were associated with greater gains in body weight, BMI and lean body mass (33). In considering all these studies, it is important to note that “*return to health*” weight gains were mostly modest.

Therefore, substantial evidence supports a phenomenon of “*return to health*” weight gain observed following cART-initiation. A limitation of all these studies is the lack of “true” baseline healthy weight measures, recognising that such measures were unfeasible or would need to rely on self-reported (and hence unreliable) data. This reset of body habitus equilibrium following effective treatment of a catabolic infection requires distinguishing from excessive weight gain and specific body fat partitioning disorders, such as lipodystrophy.

There is evidence, however, that the weight trajectory after cART initiation can exceed that of “*return to health*” (Figure 1) in studies reporting weight changes but in distinctly different circumstances: in populations where the global obesity epidemic has already impacted and where cART is initiated much earlier in the history of HIV-infection and its catabolic effects are either modest or absent. For example, in a Texan study of 1,214 participants who were predominantly Hispanic cART-recipients, a significant weight gain (defined as greater than a 3% annual BMI increase) was observed in 24% of the cohort (28). In the large North American AIDS Cohort Collaboration on Research and Design (NA-ACCORD) study (*n* = 14,084), participants had a modest weight increase after 1 year of cART (mean 1.4 kg), including a mean 5 kg weight gain in those initially underweight (20). However, in both cohorts, there appeared to be individuals susceptible to far greater weight gains, with 12–18% transitioning to overt obesity (20, 28). These studies are limited by their reliance on weight and BMI, both of which fail to measure differences in body fat and lean muscle mass and make no estimate of central obesity, a significant determinant of cardiometabolic health and risk. In considering the metabolic impact of weight gain, it is important to consider central obesity and the development and metabolic consequences of lipodystrophy which was associated with earlier cART regimens.

## CART-RElated lipodystrophy, weight gain , and metabolic disturbances

Several studies on North American cohorts have demonstrated that following cART-initiation, weight gain typically occurs in the first 1–2 years (16, 20, 28, 29) and plateaus over time (20). However, studies reporting post-cART weight gain which controlled for markers of HIV disease progression such as AIDS diagnosis, CD4^+^ counts and HIV viral RNA concentration, have shown conflicting results. Two related large-scale studies of people living with HIV-infection (one retrospective study of participants with mean 11 years duration of HIV-infection and another 20-year prospective study) failed to demonstrate an association between cART therapy and longitudinal weight gain (21, 22). In another study, 38 patients from the Nutrition For Life (NFL) cohort from the US were followed for a mean 12 months after initiating a PI-based cART regimen and experienced a mean increase in weight (1.54 kg) and BMI (0.50 kg/m^2^) (25). Lastly, in a large cohort of 9,321 individuals, PI-based regimens were associated with a significant rise in BMI 1-year after cART-initiation compared to non-NRTI (NNRTI)-based regimens, however this was predominantly modest weight gain within the healthy-weight BMI category (34).

Conflicting results based on measures such as BMI and weight could be explained by the association between lipodystrophy and use of early cART regimens (PIs and NRTIs such as stavudine and zidovudine, which are no longer recommended) (35). Studies have demonstrated different mechanisms that adversely affect adipocyte metabolism and viability. Both PIs and NRTIs inhibit DNA polymerase-γ leading to depletion of adipocyte mitochondrial DNA and hence mitochondrial toxicity (36–39). Depleted biologically active peripheral adipocytes are associated with increased circulating free fatty acids and selective uptake by and deposition in the visceral/central adipose tissue, leading to cART-associated central abdominal and dorsocervical obesity (36). PIs induce reduced expression of cytoplasmic retinoic acid-binding protein type 1 (CRABP1) in peripheral adipocytes, thereby increasing apoptosis and reducing proliferation or replacement of peripheral adipocytes. PIs also down-regulate peroxisomal proliferator-activator receptor γ (PPAR-γ), a nuclear transcription factor essential in adipocyte differentiation and function (39), thus promoting peripheral lipolysis and inhibition of peripheral lipogenesis (37). PIs have also been associated with impaired release of metabolically important adipokines, such as adiponectin (36), which regulates hepatic lipid metabolism genes involved in lipogenesis and cholesterol synthesis and transport, enhances skeletal muscle fatty acid oxidation and transport, and has anti-inflammatory and anti-oxidative properties (40). Serum adiponectin concentrations are inversely correlated with central abdominal obesity and metabolic syndrome in people living with HIV-infection (41–43).

More recent cART regimens appear to carry comparatively modest risk of lipodystrophy compared to earlier cART therapy, however detailed data are lacking and clinical observations may be obscured by the modern obesity epidemic. Studies have shown that the second-generation NRTI festinavir is 100 times less toxic to mitochondrial DNA polymerase-γ in adipose tissue compared to the older NRTI stavudine (36). A relatively newer class of antiretrovirals, the integrase inhibitors, appear to have a neutral or inferior effect: raltegravir has recently been shown to have no adverse effect on adipocyte differentiation and adipokine secretion, while elvitegravir induced less reduction in adipocyte expression of PPAR-γ, lipoprotein lipase and adiponectin, compared with efavirenz, a first-generation NRTI (44). However, a recent systematic review of limited available RCTs evaluating lipodystrophy risk with newer PIs (atazanavir and darunavir) and raltegravir showed neutral effects on peripheral lipoatrophy and conflicting effects on central lipohypertrophy (45). Furthermore, even the newer antiretrovirals such as integrase inhibitors and second-generation PIs and NNRTIs are reported to cause weight gain after controlling for markers of HIV disease activity and progression (46). Recent studies also showed modest BMI increases with use of additional newer antiretroviral agents, entry inhibitors maraviroc (47), and enfuvirtide (48), however did not control for severity or stage of HIV-infection. The “*return to health*” phenomenon in these studies of more modern antiretroviral agents (46–48) cannot be excluded.

## Weight gain, cart, and the modern obesity trajectory: the obesogenic environment

In reviewing the potential effects of cART initiation on weight, it is important to consider the impact of existing in the modern obesogenic environment. People living with treated HIV-infection today enjoy greater life expectancy and thus are more exposed to the obesogenic environment and accumulate age-related cardiometabolic risk factors (3, 4). For example, the metabolic syndrome (the clustering of central obesity, dyslipidaemia, hypertension and insulin resistance) occurs in 17–24% of people living with treated HIV and its prevalence increases with age (49, 50).

Relatively recent data show that people living with HIV-infection have rates of overweight and obesity similar to that observed in the uninfected population (16, 20–22, 28, 51). US data from 2000 onwards show high rates of overweight and obesity between 40 and 63% in people living with HIV-infection both pre-cART and on cART (21, 22, 28, 51), with no sparing of youth (52) and paralleling US obesity statistics in the general population (21, 28, 51). In contrast to earlier studies on cART initiation in the late Twentieth century where body habitus at HIV diagnosis was characterised by cachexia, recent US data (16, 21) show that 44–54% of people initiating cART are either overweight or obese, prompting authors to raise the question, “where did all the wasting go?” (16).

There are mixed modern data on the degree of weight gain after cART-initiation and prevalence of transition into deleterious categories of excess weight. One study reported that 20% of participants with normal baseline weight became overweight or obese at 24 months post cART-initiation (16). As further evidence of the “*return to health*” weight gain described above, low baseline CD4^+^ count (<50 cells/μL) was a significant risk factor for BMI gain at 6- and 24-months (16). In contrast, the NA-ACCORD study of 14,084 participants reported pre-cART obesity at lower rates increasing from 9 to 18% between 1998 and 2010, however 22% transitioned from normal-weight to overweight and 18% from overweight to obese post cART-initiation (20). These pre-cART differences may be explained by differences in obesity determinants within the North American population, such as geography, ethnicity, education and socioeconomic status. For example, a majority Hispanic, Texan population observed between 2007 and 2010 had pre-cART prevalence of overweight and obesity of 59.6% (compared to 66.3% of the general southern Texan adult population) and 11.8% transitioned to obesity during follow-up (28).

These studies demonstrate the elevated and rising overweight and obesity rates in untreated and treated HIV-infection in well-resourced settings such as North America which has highly prevalent obesity rates. It is concerning that there is a lack of strategies or guidelines to prevent excessive weight gain in individuals initiating cART, particularly those already overweight or obese, to abrogate the weight gain that has been so frequently documented with cART.

## Weight gain during HIV treatment: a double-edged sword?

Weight gain and obesity are well-documented risk factors in the HIV-uninfected population for cardiometabolic conditions such as diabetes and CVD, in addition to premature mortality (53–56). Excess weight in cART-recipients may, however, be a double-edged sword. Overweight and obesity during HIV treatment increases the risk of developing diabetes (13) and CVD (57), however greater weight has also been associated with more effective virological suppression, higher CD4^+^ counts, slower disease progression and decreased mortality (27, 58–65). The question of how to best manage weight gain during HIV treatment is therefore challenging yet of great clinical relevance especially in the current climate of earlier and more prevalent cART-initiation, increasing overweight and obesity and growing burden of associated metabolic and cardiovascular disease in people living with HIV-infection. However, studies examining the effects of weight gain in HIV-infected cART-recipients on the risk of incident glucose disorders (pre-diabetes and diabetes), CVD and mortality are currently limited. The literature reporting the relationship between interval weight or adiposity gain during cART and the risk of incident glucose disorders, CVD and mortality, was therefore reviewed. Most reports are from well-resourced nations with early access to cART and with high rates of obesity, unless stated otherwise.

Three studies have highlighted that longitudinal increases in adiposity, whether measured by central adiposity (66), total weight (67), or BMI (34), increase the risk of incident diabetes. Only one study has specifically examined CVD risk and found BMI gain increased CVD risk, although the relationship was dependent on pre-treatment BMI (34). One study in a well-resourced setting (17) and three studies in resource-poor settings (68–70) showed short-term mortality benefits associated with weight gain during cART treatment, especially in those who were underweight or cachectic prior to cART-initiation. These studies are summarised in Table 1 and are examined in further detail below. A limitation of all but one (67) of these studies was the lack of weight variation measures in population controls.

**Table 1:** Longitudinal observation studies reporting weight gain during chronic treatment of HIV-infection with incident outcomes of diabetes, cardiovascular disease, and mortality.

**Incident outcome**	**Author**	**N**	**Cohort**	**Cohort body weight at baseline**	**Weight variable**	**Result**	**Obesogenic environment present**	**“*Return to health*” phenomenon present**
Diabetes	Herrin et al. (67)	7,177	VACS	4.6%—underweight 49.6%—normal-weight 32.4%—overweight 13.5%—obese	Weight gain (kg)	14% increased risk of diabetes for every 2.3 kg of weight gained in HIV-positive 8% in HIV-negative	Yes	Yes
Diabetes	Achhra et al. (34)	9,193	D:A:D	≈ 6.1%—underweight ≈ 64.3%—normal-weight ≈ 23.2%—overweight ≈ 6.3%—obese	BMI gain (kg/m^2^)	12% increased risk of diabetes for every 1.0 kg/m^2^ of BMI gained 2.6-fold increased risk of diabetes with highest quartile BMI gain	Yes	No
Diabetes and pre-diabetes	McMahon et al. (66)	104	Australian	Mean 24.3 ± 2.6 kg/m^2^ (BMI)	Total body fat, abdominal fat (kg, %)	12-month (HR 2.65) and 2–4 year (HR 3.16) abdominal fat gain were independently associated with incident diabetes and pre-diabetes	Yes	No
CVD	Achhra et al. (34)	9,321	D:A:D	6.1%—underweight 64.3%—normal-weight 23.2%—overweight 6.3%—obese	BMI gain (kg/m^2^)	18–20% increased risk of CVD for every 1.0 kg/m^2^ of BMI gained in initially normal-weight 10% reduced risk in initially underweight No change in risk in initially overweight-obese	Yes	No
5-year mortality	Yuh et al. (17)	4,311	VACS	5.7%—underweight 51.6%—normal-weight 30.2%—overweight 12.5%—obese	Weight gain (kg)	Weight gain >2.3 kg reduced 5-year mortality in those initially underweight or normal-weight	Yes	Yes
Early mortality	Madec et al. (68)	5,069	Kenya, Cambodia	39.3%—underweight 18.9%−18.5–20 kg/m^2^ 35.1%—>20 kg/m^2^	Weight gain (%)	Weight gain >10% at 3- and 6-months reduced mortality in those initially underweight	No	Yes
Early mortality	Sudfeld et al. (69)	3,389	Tanzania	27.3%—underweight 57.8%—normal-weight 14.9%—overweight-obese	Total body weight gain (%)	Weight loss at 1-month increased mortality in all BMI categories especially in those initially underweight	No	Yes
Early mortality	Koethe et al. (70)	27,915	Zambia	29.5%—underweight 70.5%—≥18.5 kg/m^2^	Total body weight gain (%, kg)	Weight gain ≥10 kg and ≥20% at 6-months in those initially underweight produced greatest survival benefit	No	Yes

*HIV, human immunodeficiency virus; CVD, cardiovascular disease; BMI, body mass index; HR, hazard ratio. Underweight = BMI < 18.5 kg/m^2^; Normal-weight = BMI 18.5– < 25.0 kg/m^2^; Overweight = BMI 25.0– < 30.0 kg/m^2^; Obese = BMI ≥ 30.0 kg/m^2^. VACS cohort = >40,000 HIV-infected armed forces veterans from US Department of Veteran Affairs national databases, uniquely predominantly male African-Americans. D:A:D cohort = >49,000 people living with HIV from US, Australia and European countries reflecting a modern cART-treated population from well-resourced countries. Obesogenic environment present: Suggested by highly prevalent overweight and obesity at baseline in people living with HIV, reflective of societal trends in the general population. ‘Return to health' phenomenon present: Indicated that the study cohort included participants with baseline features consistent with advanced catabolic disease*.

## The impact of weight gain on diabetes risk in cart-recipients

Glucose disorders are prevalent in treated HIV-infection (71, 72). Studies that have reported the association between incident glucose disorders and weight gain in treated HIV-infection are summarised in Table 1. A recent meta-analysis (13) of 44 studies of cART-recipients published between 2000 and 2017 showed pooled incidence rates for pre-diabetes and diabetes of 125/1000 and 13.7/1000 person-years of follow-up (PYFU), respectively. Substantial incidence heterogeneity was evident between studies, likely due to differences in participant demographics, cART regimens and duration, and diagnostic criteria for diabetes. However, many of these studies likely underestimated diabetes incidence, for various reasons. For example, HbA1c may underestimate glycaemia in treated HIV-infection due to cART effects on erythrocyte dynamics and haemolysis, which reduce the erythrocyte life span, thereby lowering HbA1c (73). A second factor is the lack of generalised use of the diagnostic 75 g OGTT which will often detect diabetes missed by fasting glucose (66) and/or HbA1c measurements (74) in cART-recipients. A recent study found that the 75 g OGTT identified an extra case burden of 54% of pre-diabetes and 11% of diabetes cases missed by fasting plasma glucose measurements (66). A further study has confirmed the utility of the OGTT which diagnosed 5.9% of diabetes cases in 220 cART-recipients, compared to 3.2% using fasting glucose and no cases with HbA1c (75).

Risk factors for diabetes in treated HIV-infection include traditional risk factors such as older age, family history, overweight/obesity, central obesity, and specific cART-related factors, including lipodystrophy, and dyslipidaemia (13). Elevated serum triglycerides and lipodystrophy were independent predictors for incident diabetes in a large cohort of 16,632 people living with HIV-infection from the D:A:D study (76). HIV-infection itself can contribute to insulin resistance and diabetes risk through lipodystrophy, systemic inflammation and interference with adipokine signalling (72, 77, 78). Certain cART exposures have been reported to be associated with increased diabetes risk, including the PIs indinavir, ritonavir, atazanavir, the NRTIs zidovudine, didanosine, lamuvidine, stavudine, and the NNRTIs efavirenz and nevirapine. However, results from studies included in the recent meta-analysis have been conflicting (13). Detailed description of the mechanisms implicated between cART and impairments in both insulin sensitivity and secretion are outside the scope of this review, however interested readers are referred to an elegant and detailed review (79). Briefly, DNA polymerase-γ, the enzyme responsible for mitochondrial DNA replication, is inhibited by NRTIs, leading to mitochondrial DNA and enzyme depletion and mitochondrial dysfunction (80). NRTIs also reduce expression of adipose mitochondrial RNA and nuclear genes involved in lipid metabolism which can result in fat deposition, insulin resistance and interference with glucose uptake in skeletal muscle (15). Further, PIs inhibit peripheral insulin-mediated glucose uptake (81–83) by non-competitive inhibition of the insulin-responsive glucose transporter GLUT-4 (84, 85). HIV-infection- and cART-related factors contributing to insulin resistance and diabetes in people living with HIV-infection have been documented in further detail elsewhere (71, 72, 77, 78).

Three studies, all published in the last 3 years, have reported on the association of interval weight gain during HIV treatment and risk of incident glucose disorders. The St Vincent's HIV and Diabetes Study observed a cohort of 104 men with treated HIV-infection for up to 18 years (mean 11 years) (66). At baseline, the cohort had a mean age of approximately 43 years, BMI 24.3 kg/m^2^, and HIV-infection duration of 8.4 years (66). A large proportion had lipodystrophy, reflecting exposure to earlier antiretroviral medications. All participants were non-obese. Pre-diabetes was defined as two fasting plasma glucose concentrations of 5.6–6.9 mmol/L and/or 2-h plasma glucose 7.8–11.0 mmol/L following an OGTT. Overt diabetes was defined as two fasting plasma glucose concentrations ≥7.0 mmol/L and/or 2-h plasma glucose ≥11.1 mmol/L after an OGTT, or physician-diagnosed diabetes during follow-up. Men with incident glucose disorders had similar baseline mean BMI to men who did not (24.4 ± 2.7 kg/m^2^ vs. 24.3 ± 2.4 kg/m^2^). In a subgroup of 58 men, longitudinal measures of total body and central abdominal fat using dual-energy X-ray absorptiometry (DEXA) revealed a positive association between incident glucose disorders and central abdominal fat gain at both 12 months and 2–4 years (66). Alternatively, no association was found with interval change in BMI, weight or total body fat. In multivariate cox-regression analyses, 12-month abdominal fat gain was independently associated with a 2.65-fold increased risk of incident glucose disorders in the long-term, whereas 2–4 years abdominal fat gain carried a 3.16-fold increased risk. This study highlighted that even modest abdominal fat gain within the normal-range of BMI appears to increase risk of developing pre-diabetes and diabetes in men living with treated HIV-infection, an association which may not be appreciated by measuring BMI or weight alone.

The second study, using data from the Veterans Aging Cohort Study (VACS) (67), examined whether weight gained during the first year after cART-initiation was associated with incident diabetes and if this association was similar in HIV-uninfected, demographically matched controls. Incident diabetes was defined as an HbA1c value ≥6.5%. Weight gain exceeding 2.3 kg following cART-initiation occurred more commonly and to a greater degree in the cohort with treated HIV-infection (47.8% with median weight gain of 2 kg) compared to controls (31.4% with median weight gain of 0.5 kg). Both groups showed a positive and linear association between weight gain ≥2.3 kg and incident diabetes with a steeper slope of association in people living with HIV-infection. For every 2.3 kg of weight gained, cART-recipients had a 14% increased risk of diabetes compared to an 8% increase in controls. This study demonstrated three key factors: (i) weight gain was common following cART-initiation; (ii) even modest weight gain in cART-recipients increased risk of incident diabetes; and (iii) weight gain in cART-recipients conferred greater risk of diabetes than in the HIV-uninfected population.

The third study, the D:A:D cohort study (34), assessed impact of BMI gain 52 weeks post-cART-initiation on incident diabetes risk. Diabetes was ascertained by either two fasting glucose concentrations >7.0 mmol/L, HbA1c >6.5%, random glucose >11.1 mmol/L with symptoms of hyperglycaemia, 2-h glucose concentration >11.1 mmol/L following an OGTT, or use of anti-diabetic medication. Incidence rates (/1000 PYFU) for diabetes by baseline BMI category were 2.04 in the initially underweight, 2.01 in the normal-weight, 4.05 in the overweight and 9.97 in the obese. In fully adjusted models, each 1.0 kg/m^2^ gain in BMI was associated with a 12% increased risk of incident diabetes, and the highest quartile of BMI gain was associated with a 2.6-fold increased risk of incident diabetes, regardless of the pre-cART BMI category.

The VACS (67) and D:A:D (34) studies used measures of weight and BMI, respectively to describe changes in body weight, whereas the St Vincent's HIV and Diabetes Study (66) also utilised direct measures of abdominal fat and body fat distribution. However, when considered together, the existing data suggest that gains in adiposity on cART, increase the risk of incident glucose disorders (Table 1). Further detailed studies are awaited.

## The impact of weight gain on CVD risk in CART-recipients

Whilst the introduction of cART has been associated with dramatically reduced all-cause mortality and AIDS-related death rates in people living with HIV-infection (86–91), rates of CVD (92, 93) and the proportion of deaths related to CVD have been rising in people living with treated HIV-infection (86, 94). It is important to consider that reports of increased incidence of diabetes and CVD in treated HIV-infection were published even prior to today's obesity epidemic (71, 72, 95). In part, this may be attributed to longer life expectancy and thus increased exposure to age-related CVD risk factors such as lifestyle factors, weight gain, overweight/obesity, hypertension, dyslipidaemia and diabetes. The D:A:D study group assessed 33,347 people living with HIV-infection with a total 160,000 PYFU and confirmed advanced age, prior CVD history, cigarette smoking, dyslipidaemia, lipodystrophy, and diabetes were all risk factors for CVD in HIV-infection in a duration-dependent manner (96).

Several large-scale analyses have also demonstrated that people living with HIV-infection are at higher risk for incident CVD events than those without, with incidence risk ratios ranging between 1.50 and 2.45 (97–101) after adjusting for age and sex and 1.21–1.93 (97, 100–102) after controlling for traditional CVD risk factors. Two studies have shown that the increased relative CVD risk among people living with HIV-infection compared to the general population is even greater in women than in men: 2.98- vs. 1.40-fold with CVD-risk factor adjustment (97) and 2.7- vs. 1.4-fold in demographically adjusted analyses (98), respectively. Further, when compared within age groups, people living with treated HIV-infection aged 18–44 years have higher relative CVD risk compared to non-infected controls than in the 45 years or older age bracket (98, 102, 103). Some cART-treated populations may also possess a higher CVD risk profile since higher prevalence rates of cigarette smoking and dyslipidaemia have been described, as well as, higher CVD rates than their non-infected peers, even when leaner (104, 105).

The studies reporting CVD during HIV treatment must be viewed carefully, however, as there is some potential for bias: in some populations, people living with HIV-infection have more frequent contact with the health system and may have more opportunity for screening and CVD diagnosis. Nevertheless, the above studies are consistent with increased CVD risk in people living with treated HIV-infection, relative to the general population. This risk appears to be even greater in younger and female populations and is not fully explained by traditional CVD risk factors, suggestive that HIV-infection or cART-related factors may be involved. Potential HIV-specific mechanisms include HIV disease progression (106, 107), inflammation and altered coagulation (108), impaired arterial elasticity (109), and endothelial dysfunction (110). HIV itself also increases risk of atherosclerosis by several mechanisms contributing to dyslipidaemia, lipodystrophy and hence diabetes including interference with cholesterol transport in human macrophages and stimulation of pro-inflammatory cytokine release, e.g., TNFα which impairs free fatty acid metabolism and lipolysis (36). Further, cART-related side effects previously mentioned including lipodystrophy and insulin resistance contribute to increased diabetes and CVD risk (12–15). CART contributes to dyslipidaemia by interfering with hydrolysis of lipase and triglyceride-rich lipoproteins, post-prandial free fatty acid and lipoprotein catabolism and peripheral fatty acid uptake (36). Cumulative PI exposure (indinavir, ritonavir, lopinavir) has been shown to be an independent CVD risk factor, whereas NRTI-related CVD risk may be dependent on traditional risk factors (111–113). PIs have historically been most associated with the greatest disturbances in lipid metabolism, with implicated mechanisms including direct binding with lipid metabolism proteins such as CRABP1 (which promotes peripheral lipoatrophy) and low-density lipoprotein-receptor-related protein type 1 (thereby increasing plasma triglyceride-rich lipoproteins) (36). Interested readers are referred to a detailed review of mechanisms whereby cART induces dyslipidaemia (36).

Excess adiposity has also been shown to be a risk factor for CVD in people living with treated HIV-infection, whether measured by elevated waist-hip ratio or BMI (114–116), and is associated with CVD risk factors such as diabetes, hypertension and dyslipidaemia (13, 117). Despite this elevated risk, only one study has examined the relationship between longitudinal weight gain during HIV treatment and subsequent risk of CVD (34). The D:A:D study reported the impact of BMI gain 52 weeks post-cART-initiation in cART-naïve individuals on the risk of incident CVD (34). An incident CVD event was defined as one or more of myocardial infarction, sudden cardiac death, an invasive coronary procedure or stroke. The heterogeneous, time-updated D:A:D cohort consists of >49,000 people living with HIV-infection from the US, several European countries and Australia; generalisable to a modern, well-resourced, cART-treated population. Characteristics at enrolment in this subset included mean age 39.6 years, 75.2% men, 33.7% white, and median BMI 23 kg/m^2^. More than 40% smoked, 2.2% had diabetes, the median CD4^+^ count was 254 cells/μL and 12.1% had a prior AIDS-defining event. This analysis investigated 9,321 people with a median follow-up of 5.3 years and found a CVD event incidence rate of 2.21/1000 PYFU. The analyses showed differing associations between BMI gain after cART-initiation and incident CVD risk, based on the pre-cART BMI. At baseline, 6.1% of participants were underweight, 64.3% normal-weight, 23.2% overweight and 6.3% obese. Participants with normal pre-cART BMI experienced 18–20% increased relative risk of CVD per 1.0 kg/m^2^ BMI gain, which was slightly attenuated after adjusting for traditional cardiovascular risk factors. In contrast, in participants who were underweight pre-cART, the relative risk of incident CVD for every 1.0 kg/m^2^ BMI gain was reduced by 10%. Interestingly, participants who were overweight or obese pre-cART did not show any change in CVD risk with every 1.0 kg/m^2^ BMI gain. This somewhat paradoxical finding may be explained by the lower number of individuals who were overweight and obese pre-cART compared to those who were normal-weight. Nevertheless, this study supports that short-term BMI gain following cART-initiation in normal-weight individuals with HIV-infection is a modest, independent risk factor for incident CVD.

Although only one study has examined the relationship between weight gain during cART and subsequent CVD risk (34), previously mentioned studies have shown that rising adiposity increases diabetes risk (34, 66, 67), which is a known risk factor for CVD in HIV-infection (96). Further studies are warranted in assessing risk of incident CVD with weight gain during HIV treatment, based on pre-cART weight, particularly when using contemporary cART regimens.

## The impact of weight gain on mortality risk in CART-recipients

Obesity and weight gain are associated with increased risk of all-cause mortality in the general population (53–55). It is unclear whether this translates to people living with HIV-infection however, given that greater weight during HIV treatment has been associated with better disease outcomes and survival (27, 58–65) and weight loss has been described as a predictor of mortality (118–120). These apparent paradoxes may be clarified by dissecting out the influences of being underweight or cachectic, the influence of weight status *per se* (rather than absolute weight gain) and considering the timing of cART-initiation in the natural history of HIV-infection and AIDS.

A number of large-scale studies in resource-limited settings, where cART has historically been commenced much later, malnourishment rates are much higher and nutritional status might be considered insecure, have shown that short-term weight gain after initiating cART has a beneficial impact on early mortality in HIV treatment (68–70) (Table 1). For example, in the 39.3% who were initially underweight (BMI < 18.5 kg/m^2^) in a large study from Kenya and Cambodia, weight gain exceeding 10% at 3- and 6-months following cART-initiation was associated with reduced mortality compared to lesser weight gain or weight loss (68). However, weight gain in groups with baseline BMI > 18.5 kg/m^2^ had no association with mortality. In a large Tanzanian cohort (*n* = 3,389), weight loss 1-month post-cART-initiation was associated with increased mortality risk compared to those who gained weight across all baseline BMI categories, but more so in those 26.9% initially underweight (69). In a Zambian cohort (*n* = 27,915, 29.5% underweight at baseline), there was a linear association between weight gain and survival, which was stronger the lower the baseline BMI category (70). The greatest survival benefit was achieved by those initially underweight who gained ≥10 kg or ≥20% weight at 6-months post-cART-initiation. These studies suggest that a “*return to health”* weight gain has beneficial effects on survival, particularly in the underweight, during HIV treatment in the resource-limited setting, where HIV-associated wasting is more prevalent and cART-initiation typically later in the natural history of HIV-infection.

A survival benefit with weight gain has also been observed in well-resourced settings, again driven by what appears to be a “*return to health*” weight gain in the underweight. Analyses from the VACS study (*n* = 4,311 from a broader cohort of >40,000 US armed forces veterans with HIV-infection) found that weight gain 12 months after cART-initiation was associated with mortality benefits in those initially underweight (17). Characteristics specific to this cohort were: predominantly male (97%), African-American (54%) and older (mean age 47.9 years). In contrast to the above studies from resource-limited settings (68–70), only 5.7% were underweight at baseline, while 51.6% were normal-weight, 30.2% overweight and 12.5% obese. Importantly, those who were underweight and normal-weight at baseline had more advanced disease severity than the remainder of the cohort. Most participants had weight gain >2.3 kg, with a median weight gain of 2.7 kg. Baseline factors associated with weight gain exceeding 4.5 kg included being underweight (OR 1.69), having CD4^+^ count < 100 cells/μL (OR 2.63) and serum haemoglobin < 12 g/dL (OR 2.57), all markers of more advanced illness. PI-based regimens were also modestly associated with greater weight gain (OR 1.3) when taken as a continuous variable. Individuals initially underweight gained disproportionately greater weight: median 7.3 kg, compared to normal-weight (3.2 kg), overweight (1.8 kg), and obese (0.9 kg) participants. Weight gain exceeding 4.5–13.6 kg conferred survival benefit in those initially underweight or normal-weight with no association found in those initially overweight or obese, after adusting for baseline disease severity. Conversely, weight loss exceeding 2.3 kg in the 12 months post-cART-initiation was associated with an increased 5-year mortality risk across all baseline BMI ranges, including in the overweight (HR 1.70) and obese (HR 2.63). In interpreting these results, it is necessary to consider that the effects of obesity and weight gain on mortality are often delayed and strengthen over time (121) and thus the follow-up period of 5 years may be insufficient to adequately assess overall impact of weight gain on mortality. Further, the unique characteristics of the VACS cohort limit the ability to extrapolate these findings to broader groups of people and women living with HIV-infection, who represented only 3% of the study population, especially since women have been shown to gain more weight following cART-initiation than males (30). Finally, the study did not examine for potential effects of social determinants of health on the relationship between weight change and mortality risk.

Thus, the aforementioned studies (17, 68–70) demonstrated beneficial associations of weight gain in the unwell and underweight commencing cART. This needs to be distinguished from the well-known adverse effects of obesity in the general population (122). Several studies in aging populations with chronic disease such as hypertension, heart failure, CVD, diabetes and emphysema have similarly demonstrated an inverse association between excess adiposity (based on BMI) and mortality risk (17, 122–125). Further, studies in people living with HIV-infection have demonstrated that higher BMI is associated with higher CD4^+^ cell count, lower HIV viral load, reduced risk of opportunistic infections, slower progression to AIDS and reduced mortality (27, 58–65), and that weight loss is associated with accelerated disease progression contributing to increased mortality (118, 120). These studies support the notion that weight status and weight gain (or loss) should not be considered as continuums, but that the outcomes may vary depending on the stage or severity of HIV-infection and pre-cART weight status. Further, the association between higher BMI and desirable HIV-associated outcomes could be explained by differences in social determinants of health, including earlier, and greater access to cART and general health care, healthier nutrition and higher levels of education, employment, income, and social status. For example, African-American ethnicity (27, 58, 60) and illicit drug use (27, 58, 61, 65) have been associated with lower BMI in HIV-infected populations, whereas no association has been found between higher BMI and education level (27, 61) or employment vs. unemployment (65). Analyses according to income level have produced conflicting results in this regard (27, 65).

The few studies supporting survival benefits of weight gain during HIV treatment (17, 68–70) have not, however, established a cause-effect relationship. Therefore, it is unclear whether weight gain itself provides survival benefits or is simply a reflection of better social determinants of health outcomes or a manifestation of a “*return to health”* phenomenon (16), as described earlier.

Increased adipose tissue stores may be protective in people receiving treatment for HIV-infection by providing surplus energy and nutritional reserves to help preserve immune function (60) and/or to survive an acute infection or illness. During HIV-infection and AIDS-related opportunistic infections, hyper-metabolism and anorexia can deplete nutrient stores, which are associated with higher risk of opportunistic infections, disease progression and mortality in cART-recipients (126). However, studies assessing a relationship between weight gain during HIV treatment and mortality in the context of nutritional and immunological status are lacking.

A different association between adiposity and mortality during HIV treatment appears to emerge based on adipose tissue distribution, specifically central obesity. For example, greater central adiposity and reduced muscle mass have been independently associated with increased 5-year mortality in people living with HIV-infection (127). In a cohort of 922 HIV-infected participants with measures of body fat and muscle mass using magnetic resonance imaging (MRI), 5-year mortality risk was reduced in those in the highest tertile of arm skeletal muscle (OR 0.51) and leg skeletal muscle (OR 0.42). In contrast, 5-year mortality risk was increased in those in the highest tertile of central adipose tissue mass (OR 2.12) (127). Increased waist circumference was also independently associated with increased mortality risk. BMI was increased by central adiposity and decreased by muscle wasting, both factors that increased mortality risk in this HIV-infected population, demonstrating the limitations of BMI and total body weight measures as risk factors for mortality in this population.

## Conclusion

The introduction of cART and more effective and earlier management strategies in the treatment of HIV/AIDS has caused a shift away from weight loss and wasting observed in the early history of HIV-infection, to weight gain, overweight and obesity in people living with HIV-infection at rates now mirroring those seen in the general population (16, 20–22).

Abdominal fat gain (66), weight gain (67), and rising BMI (34) in the short-term during HIV treatment has been shown to increase the risk of incident glucose disorders. Rising BMI is associated with increased incidence of CVD, however the relationship is dependent on pre-cART BMI category (34). Early weight gain after cART-initiation is associated with short-term survival benefit, especially in those initially underweight (17, 68–70), consistent with the health benefits of resolving a catabolic viral illness.

The rising use of cART in the aim of eradicating AIDS and the global obesity epidemic will intersect and likely accelerate the growing issues of weight gain, obesity, diabetes and CVD in people living with treated HIV-infection in coming years. These factors need to be distinguished from the important restoration of body nutrient stores in the “*return to health*” following cART-initiation. The latter may become less prevalent as initiatives to commence cART at diagnosis become generalised worldwide and fewer people experience the wasting effects of advanced HIV-infection and AIDS prior to cART-initiation.

There are insufficient data to reliably guide decision-making for clinicians regarding patient advice for weight gain, loss, and maintenance during HIV treatment. Research is needed to further characterise the relationship between weight gain during HIV treatment and subsequent risk of diabetes, CVD and mortality. This field requires comprehensive, large-scale studies focusing on various markers of change in body mass (including BMI and total body weight) and fat distribution where possible (including body composition measures e.g., DEXA or MRI, as well, as waist-hip ratio and waist circumference). Body fat distribution should be evaluated as weight and BMI do not accurately estimate visceral or abdominal adiposity, or differentiate between fat and lean body mass. Further, weight loss due to primarily muscle mass loss may, for example, portend a different health trajectory to reduction in visceral adiposity. Studies focusing on diabetes incidence need to utilise more robust diagnostic methods targeted towards people living with HIV-infection, by ensuring the 75 g OGTT is included in the diagnostic criteria. Further, impacts of weight gain and obesity on mortality are predominantly felt in the long-term (121), therefore studies evaluating the relationship between weight gain and mortality must follow patients for a longer period. Optimally, studies would also examine the role of social determinants of health in the relationship between weight gain and better HIV disease-related outcomes.

Clinicians are encouraged to address overweight and obesity as part of cardiometabolic care for people living with HIV-infection, as they would for any individual with increased cardiometabolic risk. Future observational studies reporting the impact of weight excess on cardiometabolic disease and mortality in treated HIV-infection are awaited.

## Author contributions

SK drafted the manuscript, reviewed the manuscript and approved of the final version. KS conceived the review, reviewed the manuscript and approved of the final version.

### Conflict of interest statement

The authors declare that the research was conducted in the absence of any commercial or financial relationships that could be construed as a potential conflict of interest.

## References

[1] Sharp PM Hahn BH. Origins of HIV and the AIDS pandemic. Cold Spring Harbor Perspect Med. (2011) 1:a006841. 10.1101/cshperspect.a006841 22229120 PMC3234451

[2] UNAIDS Fact Sheet—Latest Statistics on the Status of the AIDS Epidemic (2018). Available online at: http://www.unaids.org/en/resources/fact-sheet (Accessed March 29, 2018).

[3] Lohse N. The road to success. Long-term prognosis for persons living with HIV in Denmark—Time trends and risk factors. Danish Med J. (2016) 63:B5210. 26836803

[4] Sabin CA. Do people with HIV infection have a normal life expectancy in the era of combination antiretroviral therapy? BMC Med. (2013) 11:251. 10.1186/1741-7015-11-251 24283830 PMC4220799

[5] WHO Guideline on When to Start Antiretroviral Therapy and on Pre-exposure Prophylaxis for HIV (2015). Available online at: http://www.who.int/hiv/pub/guidelines/earlyrelease-arv/en/ (Accessed March 29, 2018). 26598776

[6] UNAIDS. 90-90-90: Treatment for All (2018). Available online at: http://www.unaids.org/en/resources/909090 (Accessed March 29, 2018).

[7] Hontelez JA de Vlas SJ Tanser F Bakker R Barnighausen T Newell ML. The impact of the new WHO antiretroviral treatment guidelines on HIV epidemic dynamics and cost in South Africa. PLoS ONE (2011) 6:e21919. 10.1371/journal.pone.0021919 21799755 PMC3140490

[8] Department WHOHA New WHO Recommendations: Antiretroviral Therapy for Adults and Adolescents (2018). Available online at: http://www.who.int/hiv/pub/arv/art_key_mess.pdf (Accessed April 13, 2018).

[9] Doherty M Ford N Vitoria M Weiler G Hirnschall G. The 2013 WHO guidelines for antiretroviral therapy: evidence-based recommendations to face new epidemic realities. Curr Opin HIV AIDS (2013) 8:528–34. 10.1097/COH.0000000000000008 24100873

[10] Dutta A Barker C Kallarakal A. The HIV treatment gap: estimates of the financial resources needed versus available for scale-up of antiretroviral therapy in 97 countries from 2015 to 2020. PLoS Med. (2015) 12:e1001907. 10.1371/journal.pmed.1001907 26599990 PMC4658189

[11] Hill A Pozniak A. HIV treatment cascades: how can all countries reach the UNAIDS 90-90-90 target? AIDS (2015) 29:2523–5. 10.1097/QAD.0000000000000864 26558548

[12] Carr A Samaras K Chisholm DJ Cooper DA. Pathogenesis of HIV-1-protease inhibitor-associated peripheral lipodystrophy, hyperlipidaemia, and insulin resistance. Lancet (1998) 351:1881–3. 10.1016/S0140-6736(98)03391-1 9652687

[13] Nansseu JR Bigna JJ Kaze AD Noubiap JJ. Incidence and risk factors for prediabetes and diabetes mellitus among HIV infected adults on antiretroviral therapy: systematic review and meta-analysis. Epidemiology (2018) 29:431–41. 10.1097/EDE.0000000000000815 29394189

[14] Friis-Moller N Sabin CA Weber R d'Arminio Monforte A El-Sadr WM Reiss P. Combination antiretroviral therapy and the risk of myocardial infarction. N Engl J Med. (2003) 349:1993–2003. 10.1056/NEJMoa030218 14627784

[15] Mallon PW Unemori P Sedwell R Morey A Rafferty M Williams K. *In vivo*, nucleoside reverse-transcriptase inhibitors alter expression of both mitochondrial and lipid metabolism genes in the absence of depletion of mitochondrial DNA. J Infect Dis. (2005) 191:1686–96. 10.1086/429697 15838796

[16] Tate T Willig AL Willig JH Raper JL Moneyham L Kempf MC. HIV infection and obesity: where did all the wasting go? Antiviral Ther. (2012) 17:1281–9. 10.3851/IMP2348 22951353 PMC3779137

[17] Yuh B Tate J Butt AA Crothers K Freiberg M Leaf D. Weight change after antiretroviral therapy and mortality. Clin Infect Dis. (2015) 60:1852–9. 10.1093/cid/civ192 25761868 PMC4542664

[18] Dulloo AG Jacquet J Seydoux J Montani JP. The thrifty ‘catch-up fat' phenotype: its impact on insulin sensitivity during growth trajectories to obesity and metabolic syndrome. Int J Obes. (2006) 30 (Suppl. 4):S23–35. 10.1038/sj.ijo.0803516 17133232

[19] Mulligan K Grunfeld C Tai VW Algren H Pang M Chernoff DN. Hyperlipidemia and insulin resistance are induced by protease inhibitors independent of changes in body composition in patients with HIV infection. J Acquir Immune Defic Syndr. (2000) 23:35–43. 10.1097/00126334-200001010-00005 10708054

[20] Koethe JR Jenkins CA Lau B Shepherd BE Justice AC Tate JP. Rising obesity prevalence and weight gain among adults starting antiretroviral therapy in the United States and Canada. AIDS Res Hum Retroviruses (2016) 32:50–8. 10.1089/aid.2015.0147 26352511 PMC4692122

[21] Crum-Cianflone N Tejidor R Medina S Barahona I Ganesan A. Obesity among patients with HIV: the latest epidemic. AIDS Patient Care STDs (2008) 22:925–30. 10.1089/apc.2008.0082 19072098 PMC2707924

[22] Crum-Cianflone N Roediger MP Eberly L Headd M Marconi V Ganesan A. Increasing rates of obesity among HIV-infected persons during the HIV epidemic. PLoS ONE (2010) 5:e10106. 10.1371/journal.pone.0010106 20419086 PMC2856157

[23] Pernerstorfer-Schoen H Schindler K Parschalk B Schindl A Thoeny-Lampert S Wunderer K. Beneficial effects of protease inhibitors on body composition and energy expenditure: a comparison between HIV-infected and AIDS patients. AIDS (1999) 13:2389–96. 10.1097/00002030-199912030-00010 10597780

[24] Carbonnel F Maslo C Beaugerie L Carrat F Wirbel E Aussel C. Effect of indinavir on HIV-related wasting. AIDS (1998) 12:1777–84. 10.1097/00002030-199814000-00009 9792378

[25] Silva M Skolnik PR Gorbach SL Spiegelman D Wilson IB Fernandez-DiFranco MG. The effect of protease inhibitors on weight and body composition in HIV-infected patients. AIDS (1998) 12:1645–51. 10.1097/00002030-199813000-00012 9764784

[26] McDermott AY Terrin N Wanke C Skinner S Tchetgen E Shevitz AH. CD4^+^ cell count, viral load, and highly active antiretroviral therapy use are independent predictors of body composition alterations in HIV-infected adults: a longitudinal study. Clin Infect Dis. (2005) 41:1662–70. 10.1086/498022 16267741

[27] Jones CY Hogan JW Snyder B Klein RS Rompalo A Schuman P. Overweight and human immunodeficiency virus (HIV) progression in women: associations HIV disease progression and changes in body mass index in women in the HIV epidemiology research study cohort. Clin Infect Dis. (2003) 37 (Suppl. 2):S69–80. 10.1086/375889 12942377

[28] Taylor BS Liang Y Garduno LS Walter EA Gerardi MB Anstead GM. High risk of obesity and weight gain for HIV-infected uninsured minorities. J Acquir Immune Defic Syndr. (2014) 65:e33–40. 10.1097/QAI.0000000000000010 24121754 PMC3957274

[29] Lakey W Yang LY Yancy W Chow SC Hicks C. Short communication: from wasting to obesity: initial antiretroviral therapy and weight gain in HIV-infected persons. AIDS Res Hum Retroviruses (2013) 29:435–40. 10.1089/aid.2012.0234 23072344 PMC3581041

[30] Bares SH Smeaton LM Xu A Godfrey C McComsey GA. HIV-infected women gain more weight than HIV-infected men following the initiation of antiretroviral therapy. J Women's Health (2018) 27:1162–9. 10.1089/jwh.2017.6717 29608129 PMC6148723

[31] Mave V Erlandson KM Gupte N Balagopal A Asmuth DM Campbell TB. Inflammation and change in body weight with antiretroviral therapy initiation in a multinational cohort of HIV-infected adults. J Infect Dis. (2016) 214:65–72. 10.1093/infdis/jiw096 26962236 PMC4907416

[32] Shikuma CM Zackin R Sattler F Mildvan D Nyangweso P Alston B. Changes in weight and lean body mass during highly active antiretroviral therapy. Clin Infect Dis. (2004) 39:1223–30. 10.1086/424665 15486848

[33] Erlandson KM Kitch D Tierney C Sax PE Daar ES Tebas P. Weight and lean body mass change with antiretroviral initiation and impact on bone mineral density. AIDS (2013) 27:2069–79. 10.1097/QAD.0b013e328361d25d 24384588 PMC3966569

[34] Achhra AC Mocroft A Reiss P Sabin C Ryom L de Wit S. Short-term weight gain after antiretroviral therapy initiation and subsequent risk of cardiovascular disease and diabetes: the D:A:D study. HIV Med. (2016) 17:255–68. 10.1111/hiv.12294 26216031

[35] Falutz J. Management of fat accumulation in patients with HIV infection. Curr HIV/AIDS Rep. (2011) 8:200–8. 10.1007/s11904-011-0087-3 21739217

[36] da Cunha J Maselli LM Stern AC Spada C Bydlowski SP. Impact of antiretroviral therapy on lipid metabolism of human immunodeficiency virus-infected patients: old and new drugs. World J Virol. (2015) 4:56–77. 10.5501/wjv.v4.i2.56 25964872 PMC4419122

[37] Paruthi J Gill N Mantzoros CS. Adipokines in the HIV/HAART-associated lipodystrophy syndrome. Metabolism (2013) 62:1199–205. 10.1016/j.metabol.2013.04.014 23706880

[38] Grinspoon S Carr A. Cardiovascular risk and body-fat abnormalities in HIV-infected adults. N Engl J Med. (2005) 352:48–62. 10.1056/NEJMra041811 15635112

[39] Sattler FR. Pathogenesis and treatment of lipodystrophy: what clinicians need to know. Top HIV Med. (2008) 16:127–33. 18838747

[40] Esfahani M Movahedian A Baranchi M Goodarzi MT. Adiponectin: an adipokine with protective features against metabolic syndrome. Iran J Basic Med Sci. (2015) 18:430–42. 10.22038/ijbms.2015.4404 26124928 PMC4475650

[41] Freitas P Carvalho D Santos AC Madureira AJ Martinez E Pereira J. Adipokines, hormones related to body composition, and insulin resistance in HIV fat redistribution syndrome. BMC Infect Dis. (2014) 14:347. 10.1186/1471-2334-14-347 24958357 PMC4079215

[42] Kosmiski LA Bacchetti P Kotler DP Heymsfield SB Lewis CE Shlipak MG. Relationship of fat distribution with adipokines in human immunodeficiency virus infection. J Clin Endocrinol Metab. (2008) 93:216–24. 10.1210/jc.2007-1155 17940113 PMC2190751

[43] Morimoto HK Simao AN de Almeida ER Ueda LT Oliveira SR de Oliveira NB. Role of metabolic syndrome and antiretroviral therapy in adiponectin levels and oxidative stress in HIV-1 infected patients. Nutrition (2014) 30:1324–30. 10.1016/j.nut.2014.03.017 25280407

[44] Moure R Domingo P Gallego-Escuredo JM Villarroya J Gutierrez Mdel M Mateo MG. Impact of elvitegravir on human adipocytes: alterations in differentiation, gene expression and release of adipokines and cytokines. Antiviral Res. (2016) 132:59–65. 10.1016/j.antiviral.2016.05.013 27216995

[45] Guaraldi G Stentarelli C Zona S Santoro A. HIV-associated lipodystrophy: impact of antiretroviral therapy. Drugs (2013) 73:1431–50. 10.1007/s40265-013-0108-1 24002702

[46] Taramasso L Ricci E Menzaghi B Orofino G Passerini S Madeddu G. Weight gain: a possible side effect of all antiretrovirals. Open Forum Infect Dis. (2017) 4:ofx239. 10.1093/ofid/ofx239 29255735 PMC5727459

[47] Bigoloni A Gianotti N Spagnuolo V Galli L Nozza S Cossarini F. Long-term glucose tolerance in highly experienced HIV-infected patients receiving nucleoside analogue-sparing regimens. AIDS (2012) 26:1837–40. 10.1097/QAD.0b013e32835705dd 22739393

[48] Cooper DA Cordery DV Reiss P Henry K Nelson M O'Hearn M. The effects of enfuvirtide therapy on body composition and metabolic parameters over 48 weeks in the TORO body imaging substudy. HIV Med. (2011) 12:31–9. 10.1111/j.1468-1293.2010.00845.x 20497250

[49] Zhao H Goetz MB. Complications of HIV infection in an ageing population: challenges in managing older patients on long-term combination antiretroviral therapy. J Antimicrob Chemother. (2011) 66:1210–4. 10.1093/jac/dkr058 21421583

[50] Kirk JB Goetz MB. Human immunodeficiency virus in an aging population, a complication of success. J Am Geriatr Soc. (2009) 57:2129–38. 10.1111/j.1532-5415.2009.02494.x 19793157

[51] Amorosa V Synnestvedt M Gross R Friedman H MacGregor RR Gudonis D. A tale of 2 epidemics: the intersection between obesity and HIV infection in Philadelphia. J Acquir Immune Defic Syndr. (2005) 39:557–61. 16044007

[52] Vance DE Mugavero M Willig J Raper JL Saag MS. Aging with HIV: a cross-sectional study of comorbidity prevalence and clinical characteristics across decades of life. J Assoc Nurses AIDS Care (2011) 22:17–25. 10.1016/j.jana.2010.04.002 20471864

[53] Patel AV Hildebrand JS Gapstur SM. Body mass index and all-cause mortality in a large prospective cohort of white and black U.S. adults. PLoS ONE (2014) 9:e109153. 10.1371/journal.pone.0109153 25295620 PMC4189918

[54] Ogden CL Yanovski SZ Carroll MD Flegal KM. The epidemiology of obesity. Gastroenterology (2007) 132:2087–102. 10.1053/j.gastro.2007.03.052 17498505

[55] Flegal KM Kit BK Orpana H Graubard BI. Association of all-cause mortality with overweight and obesity using standard body mass index categories: a systematic review and meta-analysis. JAMA (2013) 309:71–82. 10.1001/jama.2012.113905 23280227 PMC4855514

[56] Reaven GM. Insulin resistance: the link between obesity and cardiovascular disease. Med Clin N Am. (2011) 95:875–92. 10.1016/j.mcna.2011.06.002 21855697

[57] Lake JE. The fat of the matter: obesity and visceral adiposity in treated HIV infection. Curr HIV/AIDS Rep. (2017) 14:211–9. 10.1007/s11904-017-0368-6 29043609 PMC5694708

[58] Shuter J Chang CJ Klein RS. Prevalence and predictive value of overweight in an urban HIV care clinic. J Acquir Immune Defic Syndr. (2001) 26:291–7. 10.1097/00042560-200103010-00013 11242203

[59] Blashill AJ Mayer KH Crane HM Grasso C Safren SA. Body mass index, immune status, and virological control in HIV-infected men who have sex with men. J Int Assoc Providers AIDS Care (2013) 12:319–24. 10.1177/2325957413488182 23719237 PMC4259246

[60] Shor-Posner G Campa A Zhang G Persaud N Miguez-Burbano MJ Quesada J. When obesity is desirable: a longitudinal study of the Miami HIV-1-infected drug abusers (MIDAS) cohort. J Acquir Immune Defic Syndr. (2000) 23:81–8. 10.1097/00126334-200001010-00011 10708060

[61] Sharma A Hoover DR Shi Q Gustafson D Plankey MW Hershow RC. Relationship between body mass index and mortality in HIV-infected HAART users in the women's interagency HIV study. PLoS ONE (2015) 10:e0143740. 10.1371/journal.pone.0143740 26699870 PMC4689347

[62] Crum-Cianflone NF Roediger M Eberly LE Vyas K Landrum ML Ganesan A. Obesity among HIV-infected persons: impact of weight on CD4 cell count. AIDS (2010) 24:1069–72. 10.1097/QAD.0b013e328337fe01 20216300 PMC2878190

[63] Koethe JR Jenkins CA Shepherd BE Stinnette SE Sterling TR. An optimal body mass index range associated with improved immune reconstitution among HIV-infected adults initiating antiretroviral therapy. Clin Infect Dis. (2011) 53:952–60. 10.1093/cid/cir606 21946189 PMC3189168

[64] Womack J Tien PC Feldman J Shin JH Fennie K Anastos K. Obesity and immune cell counts in women. Metabolism (2007) 56:998–1004. 10.1016/j.metabol.2007.03.008 17570264 PMC1939725

[65] Quach LA Wanke CA Schmid CH Gorbach SL Mwamburi DM Mayer KH. Drug use and other risk factors related to lower body mass index among HIV-infected individuals. Drug Alcohol Depend. (2008) 95:30–6. 10.1016/j.drugalcdep.2007.12.004 18243579 PMC3837518

[66] McMahon CN Petoumenos K Hesse K Carr A Cooper DA Samaras K. High rates of incident diabetes and prediabetes are evident in men with treated HIV followed for 11 years. AIDS (2018) 32:451–9. 10.1097/QAD.0000000000001709 29381559

[67] Herrin M Tate JP Akgun KM Butt AA Crothers K Freiberg MS. Weight gain and incident diabetes among HIV-infected veterans initiating antiretroviral therapy compared with uninfected individuals. J Acquir Immune Defic Syndr. (2016) 73:228–36. 10.1097/QAI.0000000000001071 27171741 PMC5023454

[68] Madec Y Szumilin E Genevier C Ferradini L Balkan S Pujades M. Weight gain at 3 months of antiretroviral therapy is strongly associated with survival: evidence from two developing countries. AIDS (2009) 23:853–61. 10.1097/QAD.0b013e32832913ee 19287299

[69] Sudfeld CR Isanaka S Mugusi FM Aboud S Wang M Chalamilla GE. Weight change at 1 mo of antiretroviral therapy and its association with subsequent mortality, morbidity, and CD4 T cell reconstitution in a Tanzanian HIV-infected adult cohort. Am J Clin Nutr. (2013) 97:1278–87. 10.3945/ajcn.112.053728 23636235 PMC3652924

[70] Koethe JR Lukusa A Giganti MJ Chi BH Nyirenda CK Limbada MI. Association between weight gain and clinical outcomes among malnourished adults initiating antiretroviral therapy in Lusaka, Zambia. J Acquir Immune Defic Syndr. (2010) 53:507–13. 10.1097/QAI.0b013e3181b32baf 19730111 PMC3749827

[71] Samaras K. The burden of diabetes and hyperlipidemia in treated HIV infection and approaches for cardiometabolic care. Curr HIV/AIDS Rep. (2012) 9:206–17. 10.1007/s11904-012-0124-x 22752405

[72] Samaras K. Prevalence and pathogenesis of diabetes mellitus in HIV-1 infection treated with combined antiretroviral therapy. J Acquir Immune Defic Syndr. (2009) 50:499–505. 10.1097/QAI.0b013e31819c291b 19223782

[73] Slama L Palella FJ Jr. Abraham AG Li X Vigouroux C Pialoux G. Inaccuracy of haemoglobin A1c among HIV-infected men: effects of CD4 cell count, antiretroviral therapies and haematological parameters. J Antimicrob Chemother. (2014) 69:3360–7. 10.1093/jac/dku295 25096078 PMC4228777

[74] Seang S Lake JE Tian F Anastos K Cohen MH Tien PC Oral Glucose Tolerance Testing identifies HIV^+^ infected women with Diabetes Mellitus (DM) not captured by standard DM definition. J AIDS Clin Res. (2016) 7:545 10.4172/2155-6113.1000545 27066296 PMC4825684

[75] Coelho AR Moreira FA Santos AC Silva-Pinto A Sarmento A Carvalho D. Diabetes mellitus in HIV-infected patients: fasting glucose, A1c, or oral glucose tolerance test—which method to choose for the diagnosis? BMC Infect Dis. (2018) 18:309. 10.1186/s12879-018-3221-7 29980190 PMC6035413

[76] Petoumenos K Worm SW Fontas E Weber R De Wit S Bruyand M. Predicting the short-term risk of diabetes in HIV-positive patients: the Data Collection on Adverse Events of Anti-HIV Drugs (D:A:D) study. J Int AIDS Soc. (2012) 15:17426. 10.7448/IAS.15.2.17426 23078769 PMC3494158

[77] Willig AL Overton ET. Metabolic complications and glucose metabolism in HIV infection: a review of the evidence. Curr HIV/AIDS Rep. (2016) 13:289–96. 10.1007/s11904-016-0330-z 27541600 PMC5425100

[78] Kalra S Agrawal N. Diabetes and HIV: current understanding and future perspectives. Curr Diab Rep. (2013) 13:419–27. 10.1007/s11892-013-0369-9 23446780

[79] Hruz PW. Molecular mechanisms for insulin resistance in treated HIV-infection. Best Pract Res Clin Endocrinol Metab. (2011) 25:459–68. 10.1016/j.beem.2010.10.017 21663839 PMC3115529

[80] Brinkman K ter Hofstede HJ Burger DM Smeitink JA Koopmans PP. Adverse effects of reverse transcriptase inhibitors: mitochondrial toxicity as common pathway. AIDS (1998) 12:1735–44. 10.1097/00002030-199814000-00004 9792373

[81] Noor MA Seneviratne T Aweeka FT Lo JC Schwarz JM Mulligan K. Indinavir acutely inhibits insulin-stimulated glucose disposal in humans: a randomized, placebo-controlled study. AIDS (2002) 16:F1–8. 10.1097/00002030-200203290-00002 11964551 PMC3166537

[82] Murata H Hruz PW Mueckler M. The mechanism of insulin resistance caused by HIV protease inhibitor therapy. J Biol Chem. (2000) 275:20251–4. 10.1074/jbc.C000228200 10806189

[83] Murata H Hruz PW Mueckler M. Indinavir inhibits the glucose transporter isoform Glut4 at physiologic concentrations. AIDS (2002) 16:859–63. 10.1097/00002030-200204120-00005 11919487

[84] Hresko RC Hruz PW. HIV protease inhibitors act as competitive inhibitors of the cytoplasmic glucose binding site of GLUTs with differing affinities for GLUT1 and GLUT4. PLoS ONE (2011) 6:e25237. 10.1371/journal.pone.0025237 21966466 PMC3179492

[85] Hertel J Struthers H Horj CB Hruz PW. A structural basis for the acute effects of HIV protease inhibitors on GLUT4 intrinsic activity. J Biol Chem. (2004) 279:55147–52. 10.1074/jbc.M410826200 15496402 PMC1403823

[86] Eyawo O Franco-Villalobos C Hull MW Nohpal A Samji H Sereda P. Changes in mortality rates and causes of death in a population-based cohort of persons living with and without HIV from 1996 to 2012. BMC Infect Dis. (2017) 17:174. 10.1186/s12879-017-2254-7 28241797 PMC5329918

[87] Martinez E Milinkovic A Buira E de Lazzari E Leon A Larrousse M. Incidence and causes of death in HIV-infected persons receiving highly active antiretroviral therapy compared with estimates for the general population of similar age and from the same geographical area. HIV Med. (2007) 8:251–8. 10.1111/j.1468-1293.2007.00468.x 17461853

[88] Weber R Ruppik M Rickenbach M Spoerri A Furrer H Battegay M. Decreasing mortality and changing patterns of causes of death in the Swiss HIV Cohort Study. HIV Med. (2013) 14:195–207. 10.1111/j.1468-1293.2012.01051.x 22998068

[89] Yang CH Huang YF Hsiao CF Yeh YL Liou HR Hung CC. Trends of mortality and causes of death among HIV-infected patients in Taiwan, 1984–2005. HIV Med. (2008) 9:535–43. 10.1111/j.1468-1293.2008.00600.x 18554309

[90] Palella FJ Jr. Baker RK Moorman AC Chmiel JS Wood KC Brooks JT. Mortality in the highly active antiretroviral therapy era: changing causes of death and disease in the HIV outpatient study. J Acquir Immune Defic Syndr. (2006) 43:27–34. 10.1097/01.qai.0000233310.90484.16 16878047

[91] Smith CJ Ryom L Weber R Morlat P Pradier C Reiss P. Trends in underlying causes of death in people with HIV from 1999 to 2011 (D:A:D): a multicohort collaboration. Lancet (2014) 384:241–8. 10.1016/S0140-6736(14)60604-8 25042234

[92] Price J Hoy J Ridley E Nyulasi I Paul E Woolley I. Changes in the prevalence of lipodystrophy, metabolic syndrome and cardiovascular disease risk in HIV-infected men. Sex Health (2015) 12:240–8. 10.1071/SH14084 27470913

[93] Rickerts V Brodt H Staszewski S Stille W. Incidence of myocardial infarctions in HIV-infected patients between 1983 and 1998: the Frankfurt HIV-cohort study. Eur J Med Res. (2000) 5:329–33. 10958765

[94] Krentz HB Kliewer G Gill MJ. Changing mortality rates and causes of death for HIV-infected individuals living in Southern Alberta, Canada from 1984 to 2003. HIV Med. (2005) 6:99–106. 10.1111/j.1468-1293.2005.00271.x 15807715

[95] Rasmussen LD May MT Kronborg G Larsen CS Pedersen C Gerstoft J. Time trends for risk of severe age-related diseases in individuals with and without HIV infection in Denmark: a nationwide population-based cohort study. Lancet HIV (2015) 2:e288–98. 10.1016/S2352-3018(15)00077-6 26423253

[96] Worm SW De Wit S Weber R Sabin CA Reiss P El-Sadr W. Diabetes mellitus, preexisting coronary heart disease, and the risk of subsequent coronary heart disease events in patients infected with human immunodeficiency virus: the Data Collection on Adverse Events of Anti-HIV Drugs (D:A:D Study). Circulation (2009) 119:805–11. 10.1161/CIRCULATIONAHA.108.790857 19188509 PMC2715841

[97] Triant VA Lee H Hadigan C Grinspoon SK. Increased acute myocardial infarction rates and cardiovascular risk factors among patients with human immunodeficiency virus disease. J Clin Endocrinol Metab. (2007) 92:2506–12. 10.1210/jc.2006-2190 17456578 PMC2763385

[98] Lang S Mary-Krause M Cotte L Gilquin J Partisani M Simon A. Increased risk of myocardial infarction in HIV-infected patients in France, relative to the general population. AIDS (2010) 24:1228–30. 10.1097/QAD.0b013e328339192f 20400883

[99] Obel N Thomsen HF Kronborg G Larsen CS Hildebrandt PR Sorensen HT. Ischemic heart disease in HIV-infected and HIV-uninfected individuals: a population-based cohort study. Clin Infect Dis. (2007) 44:1625–31. 10.1086/518285 17516408

[100] Freiberg MS Chang CC Kuller LH Skanderson M Lowy E Kraemer KL. HIV infection and the risk of acute myocardial infarction. JAMA Intern Med. (2013) 173:614–22. 10.1001/jamainternmed.2013.3728 23459863 PMC4766798

[101] Triant VA Meigs JB Grinspoon SK. Association of C-reactive protein and HIV infection with acute myocardial infarction. J Acquir Immune Defic Syndr. (2009) 51:268–73. 10.1097/QAI.0b013e3181a9992c 19387353 PMC2763381

[102] Drozd DR Kitahata MM Althoff KN Zhang J Gange SJ Napravnik S. Increased risk of myocardial infarction in HIV-infected individuals in North America compared with the general population. J Acquir Immune Defic Syndr. (2017) 75:568–76. 10.1097/QAI.0000000000001450 28520615 PMC5522001

[103] Currier JS Taylor A Boyd F Dezii CM Kawabata H Burtcel B. Coronary heart disease in HIV-infected individuals. J Acquir Immune Defic Syndr. (2003) 33:506–12. 10.1097/00126334-200308010-00012 12869840

[104] Saves M Chene G Ducimetiere P Leport C Le Moal G Amouyel P. Risk factors for coronary heart disease in patients treated for human immunodeficiency virus infection compared with the general population. Clin Infect Dis. (2003) 37:292–8. 10.1086/375844 12856222

[105] Klein D Hurley LB Quesenberry CP Jr. Sidney S. Do protease inhibitors increase the risk for coronary heart disease in patients with HIV-1 infection? J Acquir Immune Defic Syndr. (2002) 30:471–7. 10.1097/00126334-200208150-00002 12154337

[106] El-Sadr WM Lundgren J Neaton JD Gordin F Abrams D Arduino RC. CD4^+^ count-guided interruption of antiretroviral treatment. N Engl J Med. (2006) 355:2283–96. 10.1056/NEJMoa062360 17135583

[107] Lichtenstein KA Armon C Buchacz K Chmiel JS Buckner K Tedaldi EM. Low CD4^+^ T cell count is a risk factor for cardiovascular disease events in the HIV outpatient study. Clin Infect Dis. (2010) 51:435–47. 10.1086/655144 20597691

[108] Vos AG Idris NS Barth RE Klipstein-Grobusch K Grobbee DE. Pro-inflammatory markers in relation to cardiovascular disease in HIV infection. A systematic review. PLoS ONE (2016) 11:e0147484. 10.1371/journal.pone.0147484 26808540 PMC4726827

[109] Baker JV Duprez D Rapkin J Hullsiek KH Quick H Grimm R. Untreated HIV infection and large and small artery elasticity. J Acquir Immune Defic Syndr. (2009) 52:25–31. 10.1097/qai.0b013e3181b02e6a 19731451 PMC2764552

[110] Torriani FJ Komarow L Parker RA Cotter BR Currier JS Dube MP. Endothelial function in human immunodeficiency virus-infected antiretroviral-naive subjects before and after starting potent antiretroviral therapy: the ACTG (AIDS Clinical Trials Group) Study 5152s. J Am Coll Cardiol. (2008) 52:569–76. 10.1016/j.jacc.2008.04.049 18687253 PMC2603599

[111] Friis-Moller N Reiss P Sabin CA Weber R Monforte A El-Sadr W. Class of antiretroviral drugs and the risk of myocardial infarction. N Engl J Med. (2007) 356:1723–35. 10.1056/NEJMoa062744 17460226

[112] Worm SW Sabin C Weber R Reiss P El-Sadr W Dabis F. Risk of myocardial infarction in patients with HIV infection exposed to specific individual antiretroviral drugs from the 3 major drug classes: the data collection on adverse events of anti-HIV drugs (D:A:D) study. J Infect Dis. (2010) 201:318–30. 10.1086/649897 20039804

[113] Bavinger C Bendavid E Niehaus K Olshen RA Olkin I Sundaram V. Risk of cardiovascular disease from antiretroviral therapy for HIV: a systematic review. PLoS ONE (2013) 8:e59551. 10.1371/journal.pone.0059551 23555704 PMC3608726

[114] Asztalos BF Matera R Horvath KV Horan M Tani M Polak JF. Cardiovascular disease-risk markers in HIV patients. J AIDS Clin Res. (2014) 5. 10.4172/2155-6113.1000317 26005590 PMC4439003

[115] Mercie P Thiebaut R Lavignolle V Pellegrin JL Yvorra-Vives MC Morlat P. Evaluation of cardiovascular risk factors in HIV-1 infected patients using carotid intima-media thickness measurement. Ann Med. (2002) 34:55–63. 10.1080/078538902317338652 12014436

[116] Worm SW Sabin CA Reiss P El-Sadr W Monforte A Pradier C Presence of the metabolic syndrome is not a better predictor of cardiovascular disease than the sum of its components in HIV-infected individuals: data collection on adverse events of anti-HIV drugs (D:A:D) study. Diab Care (2009) 32:474–80. 10.2337/dc08-1394 PMC2646032 19056612

[117] Gelpi M Afzal S Lundgren J Ronit A Roen A Mocroft A Higher risk of abdominal obesity, elevated LDL cholesterol and hypertriglyceridemia, but not of hypertension, in people living with HIV: results from the copenhagen comorbidity in HIV infection (COCOMO) study. Clin Infect Dis. (2018) 67:579–86. 10.1093/cid/ciy146 29471519

[118] Palenicek JP Graham NM He YD Hoover DA Oishi JS Kingsley L. Weight loss prior to clinical AIDS as a predictor of survival. Multicenter AIDS Cohort Study Investigators. J Acquir Immune Defic Syndr Hum Retrovirol. (1995) 10:366–73. 10.1097/00042560-199511000-00009 7552499

[119] Wheeler DA Gibert CL Launer CA Muurahainen N Elion RA Abrams DI. Weight loss as a predictor of survival and disease progression in HIV infection. Terry Beirn Community Programs for Clinical Research on AIDS. J Acquir Immune Defic Syndr Hum Retrovirol. (1998) 18:80–5. 10.1097/00042560-199805010-00012 9593462

[120] Tang AM Forrester J Spiegelman D Knox TA Tchetgen E Gorbach SL. Weight loss and survival in HIV-positive patients in the era of highly active antiretroviral therapy. J Acquir Immune Defic Syndr. (2002) 31:230–6. 10.1097/00126334-200210010-00014 12394802

[121] Okunade AA Rubin RM Okunade AK. Delayed effects of obese and overweight population conditions on all-cause adult mortality rate in the USA. Front Public Health (2016) 4:212. 10.3389/fpubh.2016.00212 27734013 PMC5039184

[122] Hainer V Aldhoon-Hainerova I. Obesity paradox does exist. Diab Care (2013) 36 (Suppl. 2):S276–81. 10.2337/dcS13-2023 23882059 PMC3920805

[123] Blum A Simsolo C Sirchan R Haiek S. “Obesity paradox” in chronic obstructive pulmonary disease. Israel Med Assoc J. (2011) 13:672–5. 22279700

[124] Uretsky S Messerli FH Bangalore S Champion A Cooper-Dehoff RM Zhou Q. Obesity paradox in patients with hypertension and coronary artery disease. Am J Med. (2007) 120:863–70. 10.1016/j.amjmed.2007.05.011 17904457

[125] Curtis JP Selter JG Wang Y Rathore SS Jovin IS Jadbabaie F. The obesity paradox: body mass index and outcomes in patients with heart failure. Arch Inter Med. (2005) 165:55–61. 10.1001/archinte.165.1.55 15642875

[126] Weiser SD Young SL Cohen CR Kushel MB Tsai AC Tien PC. Conceptual framework for understanding the bidirectional links between food insecurity and HIV/AIDS. Am J Clin Nutr. (2011) 94:1729s−39s. 10.3945/ajcn.111.012070 22089434 PMC3226026

[127] Scherzer R Heymsfield SB Lee D Powderly WG Tien PC Bacchetti P. Decreased limb muscle and increased central adiposity are associated with 5-year all-cause mortality in HIV infection. AIDS (2011) 25:1405–14. 10.1097/QAD.0b013e32834884e6 21572308 PMC3933309

